# Obesity, inflammation, and cancer in dogs: Review and perspectives

**DOI:** 10.3389/fvets.2022.1004122

**Published:** 2022-10-03

**Authors:** Pedro H. Marchi, Thiago H. A. Vendramini, Mariana P. Perini, Rafael V. A. Zafalon, Andressa R. Amaral, Vanessa A. Ochamotto, Juliano C. Da Silveira, Maria L. Z. Dagli, Marcio A. Brunetto

**Affiliations:** ^1^Pet Nutrology Research Center, Department of Animal Nutrition and Production of the School of Veterinary Medicine and Animal Science, University of São Paulo, Pirassununga, Brazil; ^2^Veterinary Nutrology Service, Veterinary Teaching Hospital of the School of Veterinary Medicine and Animal Science, University of São Paulo, São Paulo, Brazil; ^3^Laboratory of Molecular, Morphophysiology and Development (LMMD), Department of Veterinary Medicine, Faculty of Animal Science and Food Engineering, University of São Paulo, Pirassununga, Brazil; ^4^Laboratory of Experimental and Comparative Oncology, Department of Pathology, School of Veterinary Medicine and Animal Science of the University of São Paulo, São Paulo, Brazil

**Keywords:** adipose tissue, adipokines, IGF-1, inflammation, neoplasm, overweight, resistin, tumor

## Abstract

Obesity is the most common nutritional disease in dogs, and its prevalence has increased in recent decades. Several countries have demonstrated a prevalence of obesity in dogs similar to that observed in humans. Chronic low-grade inflammation is a prominent basis used to explain how obesity results in numerous negative health consequences. This is well known and understood, and recent studies have pointed to the association between obesity and predisposition to specific types of cancers and their complications. Such elucidations are important because, like obesity, the prevalence of cancer in dogs has increased in recent decades, establishing cancer as a significant cause of death for these animals. In the same way, intensive advances in technology in the field of human and veterinary medicine (which even proposes the use of animal models) have optimized existing therapeutic methods, led to the development of innovative treatments, and shortened the time to diagnosis of cancer. Despite the great challenges, this review aims to highlight the evidence obtained to date on the association between obesity, inflammation, and cancer in dogs, and the possible pathophysiological mechanisms that link obesity and carcinogenesis. The potential to control cancer in animals using existing knowledge is also presented.

## Introduction

Obesity is characterized by abnormal or excessive accumulation of adipose tissue, which harms the well-being and health of animals (1). In humans, its prevalence has doubled since the 1980s, and approximately one-third of the population is classified as overweight or obese (2). This trend appears to extend to dogs, given that obesity is the most common nutritional disorder in companion animals (3). In the 2000s, two studies conducted in the United States and Australia showed that between 29 and 33.5% of dogs were overweight and between 5.1 and 7.6 %, were obese (4, 5). Studies conducted in Brazil and Japan revealed an increase in the obesity rate compared to previous studies, estimating between 25.9 and 39.8% were overweight and the rate of obesity was between 14.6 and 15.1% (6, 7). Furthermore, in Spain and China, two studies reported rates of obesity of 40.9 and 44.4% in dogs, respectively (8, 9). This scenario is a matter of concern, as it results in consequences such as those seen in human medicine, where higher rates of morbidity and mortality and lower life expectancies in obese people are evident (10). Moreover, obesity predisposes patients to other diseases, such as diabetes mellitus, cardiovascular diseases, hypertension (11), orthopedic disorders (12), sleep apnea (10) and certain types of neoplasias (13). Similarly, obese dogs have reduced life expectancies (14) and quality of life (15) and are more likely to develop comorbidities, such as orthopedic disorders (16, 17), cardiovascular disease (18), respiratory alterations (19), insulin resistance (20) and specific types of neoplasias (21–25).

Cancers of various types are commonly diagnosed in companion animals, and are significant causes of death in humans and dogs (26). A North American study, carried out with dogs, analyzed data from two decades of the Veterinary Medical Database; through a sampling of more than 74 thousand cases, the study concluded that neoplasia is the main cause of death in animals aged ~10 years (27). Generally, approximately 50% of tumors are malignant, and the main sites of growth are the skin, mammary glands, soft tissues, genital tract, and oral cavity, consisting primarily of epithelial, mesenchymal, and lymphoid tumors (28, 29). According to the oncological guidelines for dogs and cats published by the American Animal Hospital Association, there is an increase in the incidence of oncological cases that can be justified by the high life expectancy of small domestic animals as a result of improvements in nutritional management, disease control, vaccination, preventive veterinary medicine, and advances in clinical and diagnostic tests (30).

Neoplastic growth is the result of genetic and epigenetic alterations in cells (31). DNA damage can cause gene mutations, which affect the genomic integrity and may determine alterations in gene products and proteins (32). The main causes of DNA damage are chemical, physical, and biological agents such as viruses, hormones, genotoxic or non-genotoxic chemicals, and radiation; however, they may also be inheritable due to DNA replication and error-repair processes, and are more frequently observed with advancing of age (32, 33).

In 2000, Hanahan and Weinberg defined the hallmarks of cancer for the first time, comprising six biological capabilities acquired during the multistep development of human tumors (34). In another article published in 2011, the same authors included inflammation or inflammatory events as enabling characteristics. Designed to respond to tissue injuries, these can potentially result in the activation of multiple hallmark capabilities, favoring the so-called tumor-promoting consequences of inflammatory responses (35). In the most recent version of the series, Hanahan, 2021, reinforced the tumor-promoting role of inflammation and further incorporated additional proposed emerging hallmarks and enabling characteristics involving “unlocking phenotypic plasticity,” “non-mutational epigenetic reprogramming,” “polymorphic microbiomes,” and “senescent cells” (36).

Excess white adipose tissue results in the unregulated production of adipokines that cause damage and lead to increased secretion of pro-inflammatory cytokines (37). This state can culminate in the establishment of insulin resistance and chronic inflammation, promoting the development of tumors and increasing the incidence and malignancy of various types of cancers in obese patients (38, 39). Thus, dogs exposed to factors with carcinogenic potential that are affected by diseases such as obesity and diabetes mellitus are more likely to develop cancer, which directly affects the efficiency of oncological therapies (37).

The perception of white adipose tissue as an endocrine organ and its alterations in obese animals has allowed a deeper understanding of the pathophysiology of obesity and its predisposition to other diseases. The precise inflammatory, endocrine, and metabolic disorders triggered by obesity are synergistic mechanisms that promote carcinogenesis. This study aimed to clarify the effects of obesity in dogs and to elucidate their relationship with carcinogenesis. The most commonly used electronic databases for the search of the articles related to obesity, inflammation, and cancer in dogs. The articles gathered consist of a broad spectrum from various parts of the world. Terms used in the search bar consisted of “obesity”, “inflammation”, “cancer”, and “dogs”. The published literature collected is predominantly sourced from the online journal databases PubMed and Science Direct.

## Obesity, the adipose tissue, inflammation, and cancer: What are the links?

### Endocrine function of white adipose tissue in obese patients

**Figure 1:**
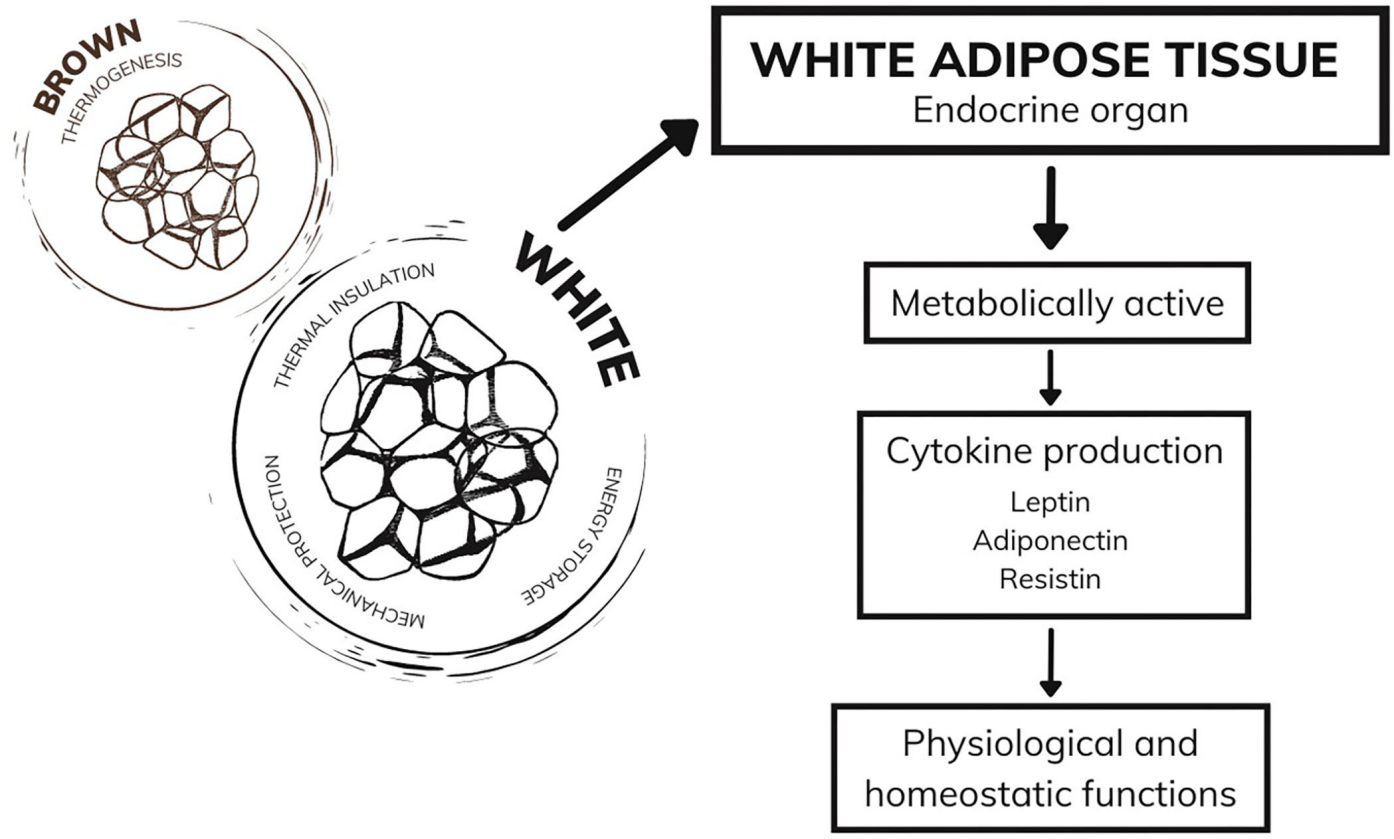
Adipose tissue and functions.

Currently considered an endocrine organ, adipose tissue is traditionally classified into two types: white adipose tissue and brown adipose tissue (40, 41). While brown adipose tissue has a function in thermogenesis, white adipose tissue, in addition to the functions of thermal insulation, mechanical protection, and energy storage, has mechanisms that act in the endocrine system (42, 43). White adipocytes are metabolically active, producing a series of hormones and cytokines responsible for regulating physiological functions, including appetite control, immune and inflammatory responses, glucose and fat metabolism, and other functions that ensure the homeostasis of the organism (44, 45). During excessive weight gain, white adipose tissue undergoes cellular and structural remodeling to adapt to the storage of many needless calories (46). Among the alterations demonstrated in humans is the expansion of adipose tissue through cellular hypertrophy (47) and remodeling of the extracellular matrix (48), which both lead to the impairment of angiogenesis and result in hypoxia, fibrosis, and inflammatory processes. Moreover, the literature suggests immune system cells infiltrate into white adipose tissue (49), which corroborates the establishment of a chronic inflammatory state. Therefore, white adipose tissue becomes dysfunctional and starts to secrete adipokines in an unregulated manner, some of which are pro-inflammatory (46) (Figure 1).

Owing to this dysfunctional expansion of white adipose tissue, obesity results in a chronic low-grade inflammatory state (50), which is characterized by the abnormal production of adipokines and pro-inflammatory cytokines, dysregulation of the inflammatory signaling cascade, and high concentrations of acute-phase proteins in the circulation (51). Concurrently, dysfunctional white adipocytes induce exaggerated recruitment of immune cells and modulate innate and adaptive immunity through adipokines (52). In a vicious cycle, excess immune cells, mainly macrophages, further stimulate and exacerbate the production and secretion of adipokines and inflammatory cytokines (46, 53), which account for several induction and regulation mechanisms of immune functions (54).

#### Pro-inflammatory cytokines

Among the pro-inflammatory cytokines secreted by white adipose tissue are interleukin (IL)-2, IL-6, tumor necrosis factor-alpha (TNF-α), C-reactive protein (CRP), and monocyte chemoattractant protein-1 (MCP-1). These cytokines act as consistent inflammatory markers in humans and are related to the development of metabolic syndromes, marked by dyslipidemia, insulin resistance, and systemic hypertension (51). These mediators are considered non-specific markers of chronic inflammation (55), and this constant low-grade inflammation has been theorized as a link to possible long-term changes in immune functions in humans (56); however, the information is quite limited for dogs.

Low concentrations of TNF-α are essential for host defense, as they limit the spread of pathogenic microorganisms into the bloodstream, triggering neutrophil adhesion, activation of cytokines, and the complement system. However, the inflammatory condition evolves with hyperstimulation. TNF-α thus assumes deleterious activity to the organism, deregulating the immune system and promoting the activation of several other cytokines, as well as the cellular oxidative system (57).

IL-6 is a cytokine with a wide range of biological activities, as it helps to control the induction of the acute phase response and is also a mediator of immunoglobulin class switching. It is also able to influence several inflammatory responses that are important in the pathogenesis of various alterations and bacterial infections, thus being considered the main regulator of the acute phase protein response (58, 59). Few studies have quantified the circulating concentrations of this adipocytokine in obese dogs; however, in most of these studies, a positive correlation was observed between increased body fat and increased circulating IL-6 (60–62).

IL-8 is a potent attractant for neutrophils. Its action is mediated by G protein-coupled CXCR1 and CXCR2 receptors (GPCRs), which have a high affinity for glutamine-leucine-arginine chemokines (63, 64). IL-8 has been detected in several human tissues affected by chronic inflammatory conditions associated with severe neutrophilic infiltration (65, 66). Endotoxins can increase IL-8 expression in neutrophils *in vitro* (67) and in horse blood when injected systemically (68). Thus, IL-8 is a key cytokine in chronic inflammatory processes and is associated with inflammatory changes. Recent studies have found decreasing concentrations of IL-8 and other ILs with weight loss in dogs (69).

Interleukin-10 (IL-10) is an important anti-inflammatory cytokine in the pathophysiology of inflammation, which attempts to counterbalance the actions of pro-inflammatory mediators, both by reducing the synthesis and release of these mediators and by antagonizing their effects. However, excessive production and release of anti-inflammatory mediators are harmful to the body's response to the invading agent, as they inhibit the release of mediators essential for the recruitment and activation of inflammatory and immune response cells (70, 71).

The literature on dogs demonstrates a correlation between obesity and chronic low-grade inflammation, based on improved inflammatory markers after weight loss in obese dogs (53, 69, 72). Its concentration has been measured before and after weight loss by several researchers, but the results are often controversial. An important study carried out by German et al. (60) described an increase in insulin resistance in obese dogs and a decrease in the concentrations of CRP and TNF-α after weight loss. Bastien, Patil, and Satyaraj (69) correlated the decrease in the concentration of pro-inflammatory cytokines like IL-2 and MCP-1 after weight loss, but the results were inconclusive in assessing IL-6 and TNF-α. Piantedosi et al. (73) also did not observe differences in the concentrations of IL-6 and TNF-α between obese dogs and ideal weight dogs; however, a more recent study by Piantedosi et al. (74) demonstrated a significant reduction in the concentrations of these adipokines in dogs subjected to a weight-loss program. Frank et al. (62) evaluated the inflammatory profile, composed of TNF-α, IL-2, IL-6, IL-8, and MCP-1, in Labrador retrievers; a correlation was described only between IL-6 and MCP-1 and increases in body condition score (BCS) of the animals. Each of the aforementioned pro-inflammatory cytokines was found at higher concentrations in obese dogs in at least one study. Despite the differences between these results, it is clear that obesity promotes an increase in the synthesis and secretion of different pro-inflammatory cytokines in dogs, leading to a state of chronic low-grade inflammation. A summary of some studies that evaluated the circulating concentration of adipokines in obese dogs and after weight loss is presented in Table 1.

**Table 1:** Summary of the main studies that evaluated adipokines in obese dogs and after weight loss.

**Year**	**Author**	**Number of animals**	**Type of obesity**	**Main results**
2002	Ishioka et al. (75)	59	Acquired	Obese dogs had higher plasma leptin concentrations than dogs with ideal body condition score (BCS).
2002	Sagawa et al. (76)	20	Induced	A positive relationship between plasma leptin concentration and body fat content in dogs.
2004	Diez et al. (77)	8	Induced	Circulating leptin concentrations correlated with body fat mass.
2004	Gayet et al. (78)	24	Induced	Plasma concentrations of insulin-like growth factor 1 (IGF-1), tumor necrosis factor (TNF)-α, and unesterified fatty acids progressively increased during the period of overfeeding.
2005	Jeussete et al. (79)	24	Induced	Obese dogs demonstrated a decrease in plasma ghrelin and an increase in leptin and insulin concentrations when compared to control dogs. During weight loss, an increase in ghrelin concentration and a decrease in leptin and plasma insulin were observed.
2007	Gayet et al. (80)	13	Induced	The plasma concentration and expression of leptin was increased, while the plasma concentration and expression of adiponectin decreased with weight gain.
2007	Ishioka et al. (81)	166	Acquired	Plasma leptin concentration was higher in dogs with higher body condition score.
2009	German et al. (60)	26	Acquired	Weight loss led to decreases in plasma concentrations of TNF- α, haptoglobin, and C-reactive protein (CRP).
2010	Brunetto, MA. (61)	10	Acquired	Positive correlation between serum leptin concentration and body fat content in dogs. Serum concentrations of triglycerides, cholesterol, interleukin (IL)-6, TNF-α, insulin, and leptin decreased after weight loss.
2011	Grant et al. (82)	9	Induced	Ad libitum feeding increased body weight, fat mass, adipocyte size, and leptin.
2012	Tvarijonaviciute et al. (72)	6	Acquired	Adiponectin concentrations increased after the weight-loss period.
2013	Van de velde et al. (83)	8	Induced	Weight gain in dogs did not change TNF-α and IL-6 concentrations.
2014	Park et al. (84)	100	Acquired	Leptin, triglycerides, and cholesterol levels were higher in the obese group. Adiponectin levels were higher in the lean group compared to the obese group.
2015	Bastien et al. (69)	18	Induced	Cytokine (monocyte chemoattractant protein (MCP)-1, IL-7, IL-2, and IL-18) concentrations decreased throughout the weight-loss program and were correlated with the percentage of fat.
2015	Frank et al. (62)	92	Acquired	Fasting plasma concentrations of IL-6 and MCP-1 were associated with an increased BCS.
2016	Piantedosi et al. (73)	40	Acquired	Obese dogs had higher serum leptin, but lower concentrations of adiponectin compared to normal weight dogs.
2017	Vitger et al. (85)	16	Acquired	Decrease in leptin in both groups (with 6 and 12 weeks of weight loss). IL-8 and MCP-1 decreased in 6 weeks and IL-8 and cholesterol in 12.
2019	Jeremias et al. (86)	20	Acquired	Serum concentrations of triglycerides, IL-2, IL-6, TNF-α, insulin, leptin, and IGF-1 decreased after the weight-loss.
2020	Vendramini et al. (87)	24	Acquired	The obese group presented increased gene expression of resistin and IL-8 when compared to the weight-loss group. In adiponectin, the obese group presented increased mRNA gene expression when compared to the weight-loss group.

Obese dogs have similar characteristics to those found in humans with obesity; thus, they can be used as animal models for the study of specific comorbidities correlated with obesity. Furthermore, adipokines in obese patients contribute to a chronic inflammatory process, modulating the immune system and altering the action of insulin. These general alterations can cause predisposition to specific types of tumors (88–90).

#### Leptin

Leptin, an important adipokine, is predominantly produced by white adipocytes (91). However, leptin mRNA is detectable in other tissues, indicating that leptin can be produced at lower levels by other organs (42, 92, 93). The main function of leptin is in the satiety regulatory center located in the hypothalamus, where it binds to Ob-R receptors, stimulating anorectic neurons and suppressing orexigenic neurons (91, 94, 95). However, its receptors are present in several organs of the body, which characterizes the pleiotropic action of this hormone (96, 97). Thus, in addition to appetite control, leptin also acts on the neuroendocrine and cardiovascular systems, insulin sensitization, hematopoiesis, and immune response (98–101).

Its physiological functions are essential to guarantee the homeostasis of the organism. However, as it is produced mainly by white adipocytes, obesity causes dysregulation to its concentration and biological activity (96). In the human body, serum leptin, is directly proportional to body mass index (96, 102), with elevated levels being termed hyperleptinemia (103). Metabolic complications caused by dysfunctional white adipocytes lead to the development of leptin resistance through the interruption of leptin signaling to the hypothalamus and the alteration of leptin transport across the blood-brain barrier (54). Jung and Kim (104) reported that the inflammatory response in the hypothalamus, neuronal endoplasmic reticulum stress, and defective autophagy induce the expression of leptin-resistant factors.

Hyperleptinemia, associated with the development of resistance to its functions, is correlated with disturbed immune responses, as leptin is responsible for activating and recruiting macrophages into adipose tissue (101, 105). Thus, this hormone is intrinsically linked to the establishment of a chronic low-grade inflammatory state. Moreover, hyperleptinemia has already been associated with the development of some illnesses in humans, such as cardiovascular diseases, acute pancreatitis, hepatocellular and renal carcinomas, and lung, prostate, colorectal, and breast cancers (106–109). The relationship between leptin and breast cancer, for example, occurs through numerous mechanisms, some of which demonstrate its action in signaling pathways, such as JAK/STAT3 and MAPK, which are associated tumor development by potentiating aromatase and estrogen signaling and activity, contributing to mammary epithelial cell growth (109, 110).

In dogs, obesity appears to induce excessive production of leptin, as observed in humans (69, 74, 79). Piantedosi et al. (74) evaluated a group of 11 obese dogs using leptin levels measured before and after a weight-loss program. While overweight, the dogs had an elevated concentration of serum leptin, and after 6 months of weight loss, the levels were reduced considerably. Similarly, Bastien, Patil, and Satyaraj (69) measured the serum leptin concentration of 18 obese Beagles, demonstrating reduced leptin levels after weight loss, consistent with previous studies. Hyperleptinemia appears to predispose obese dogs to cardiac diseases (111, 112), and two studies showed high serum leptin levels in non-obese dogs correlated with diagnoses of acute pancreatitis and gallbladder mucocele (113, 114).

#### Adiponectin

Adiponectin is also a hormone secreted mainly by adipocytes and appears in the bloodstream in three different forms: low-molecular-weight, medium-molecular-weight, and high-molecular-weight multimers; the latter is the most bioactive (115). Among its functions, it has anti-inflammatory and vascular protective properties (116, 117), as well as functions to combat obesity by stimulating fatty acid metabolism and tissue sensitivity to insulin (118). In the liver, adiponectin is capable of phosphorylating and chemically interacting with several factors, sensitizing the tissue to the action of insulin and resulting in low production of endogenous glucose and low concentration of triglycerides (115, 119, 120). In addition, in skeletal muscle, owing to similar mechanisms, it results in high fatty acid metabolism and low triglyceride concentration (115, 121). Moreover, the principal mechanisms involved in the anti-inflammatory function of adiponectin are related to the suppression and modulation of macrophages and reduced expression of Toll 4 receptors, which are responsible for the generation of pro-inflammatory signals, inhibition of chemokine production, and stimulation of IL-10 production (122–124).

In humans and laboratory animals, it is widely accepted that serum adiponectin levels are inversely proportional to body mass index, which results in hypoadiponectinemia in obese conditions (125). Adiponectin has protective effects against endometrial, prostate, thyroid, and ovarian tumors (126, 127), and lower adiponectin concentrations linked to excess adipose tissue in the body can inhibit this protective function in obese patients.

In dogs, a systematic review and meta-analysis conducted by Muñoz-Prieto et al. (128) evaluated 20 different studies, the methods of which involved the measurement of serum adiponectin in obese dogs. Despite the limited amount of research available and the heterogeneity between the materials and methods, among the 20 studies evaluated, most found results were consistent with those observed in humans, in which the concentration of adiponectin was low in obese patients and increased after weight loss. One of these studies, carried out by Tropf et al. (18), subjected 46 healthy, 29 obese, and 17 ideal weight dogs to metabolic and cardiac function evaluations. From this study, it was evident that obese dogs had dyslipidemia, insulin resistance, and reduced adiponectin concentration, among other cardiac and metabolic alterations compared with healthy and ideal weight dogs. Thus, in contrast to leptin, obesity appears to have an inverse relationship with adiponectin levels in obese dogs.

#### Resistin

One of the least discussed adipokines in veterinary medicine, whose knowledge is still scarce in human medicine, is resistin. This molecule was first described by Steppan et al. (129) as a hormone synthesized by adipocytes and identified in rodents and humans. Resistin plays an important regulatory role in glucose metabolism and insulin sensitivity in rodents (130). In humans, it coordinates several physiological processes and is associated with the secretion of immune factors, such as TNFα, IL-1, IL-6, IL-8, and IL-12, which act to trigger the inflammatory response (131).

This hormone is mainly secreted by white adipocytes; thus, the effect of resistin in obese patients may elucidate how obesity predisposes to insulin resistance, as the levels of this hormone are elevated in obese patients (129). According to the literature, resistin induces insulin resistance through insulin antagonization, which reduces glucose absorption and metabolism by adipocytes, muscle cells, and other tissues (132). Furthermore, it is associated with pro-inflammatory mechanisms, which demonstrate great relevance in metabolic, inflammatory, and autoimmune diseases (133). However, the mechanisms of its expression, regulation, secretion, and circulation are still not fully understood (134).

The function and regulation of resistin may differ from the physiological state when compared with organisms affected by obesity and diabetes, since different direct and indirect regulatory mechanisms of resistin vary in experimental models (135). According to Steppan et al. (129), the expression of the gene responsible for regulating resistin in mice varies according to sex, as high concentrations of this hormone were found in the mammary tissue of females. Thus, high serum resistin concentration has already been correlated with the degree of malignancy, staging, and occurrence of metastases in women with breast cancer (136).

### The link between obesity and cancer in humans

In human medicine, there is solid evidence that correlates obesity with the development of 13 types of cancer: endometrial, esophageal, pancreatic, and renal adenocarcinomas; hepatocellular carcinoma; meningioma; multiple myeloma; and colon, rectal, ovarian, urinary bladder, thyroid, and postmenopausal breast tumors (13). Although the role of obesity in carcinogenesis is not fully understood, recent literature has suggested a range of possible mechanisms. Among these, there is a correlation between the chronic low-grade inflammatory state and tumor microenvironment (137). This relationship exists because of the potential for secretion of numerous inflammatory factors and recruitment of immune cells, as described for both pathological processes (138). Thus, owing to its similarity to the tumor microenvironment, chronic inflammation is capable of generating an environment susceptible to tumor establishment, infiltration, and growth (138).

Related to this topic, another correlated pathway between obesity and cancer development is genomic instability (139). This was considered an enabling characteristic of cancer and refers to dysfunctions in the genome maintenance process, which leads to a higher predisposition for genomic mutations (35, 140). This instability may occur as a result of chronic inflammation, both due to the continuous exposure of DNA to the damage caused by reactive oxygen species produced excessively or by adipokines with altered concentrations and action (141, 142); or through the intensification of failures in the DNA repair process (142, 143), alterations with the potential to promote neoplastic development (141, 144). Studies linking obesity and genomic instability have shown that obese patients with endometrial cancer had a higher frequency of chromosomal abnormalities than non-obese endometrial cancer patients (145) and that obese patients with esophageal adenocarcinoma have increased markers of genomic instability (145, 146). Inflammation also has the potential to silence tumor suppressor genes and interacts with the stimulation of oncogenes and their transcription factors (147, 148). A study with mice with hepatocellular carcinoma was able to identify that the oncogenes *Carboxyl ester lipase* (Cel) gene and *Harvey rat sarcoma virus oncogene 1* (Hras) had a higher frequency of mutations in obese mice when compared to lean mice (149).

Avgerinos et al. (150) hypothesized that the main pathways present in the pathophysiology of both obesity and the development of tumors are insulin resistance, hyperinsulinemia, and abnormal action of insulin-like growth factor 1 (IGF-1); synthesis and aromatization of steroid hormones; chronic low-grade inflammatory state and oxidative stress; pathophysiological changes in adipokines; secretion of factors derived from ectopic fat deposition; microenvironment and cellular disturbances; changes in the circadian cycle and dietary nutrients; and altered gut microbiome and physical factors associated with obesity.

Hormonal carcinogenesis, in which cancers are associated with endogenous and exogenous hormones, can induce cell proliferation and eventual genetic errors, resulting in breast, endometrial, ovarian, prostatic, testicular, thyroid, cancers as well as osteosarcomas (31). Humans with obesity develop excess adipose tissue dysfunction, which results in a high concentration of estrogen in the body (151). This change in sex hormone concentration is associated with an increased risk of postmenopausal breast cancer in women (152). Furthermore, the inflammatory state induced by obesity can alter estrogen signaling, leading to DNA damage, cell proliferation, promotion of angiogenesis, and mutagenesis in several types of neoplasia (153).

Insulin resistance caused by obesity is related to the development of a chronic low-grade inflammatory state and the action of adipokines, such as leptin, resistin, TNF-α, IL-6, MCP-1, and CRP (154, 155). Consequently, the pancreas secretes a higher concentration of insulin into circulation, which suppresses the formation of IGF-1-binding proteins (156, 157). Insulin and bioavailable IGF-1 act as promoters of cell growth, angiogenesis, and lymphangiogenesis and as suppressors of apoptotic activity, mechanisms that are all considered to promote tumor development (158).

**Figure 2:**
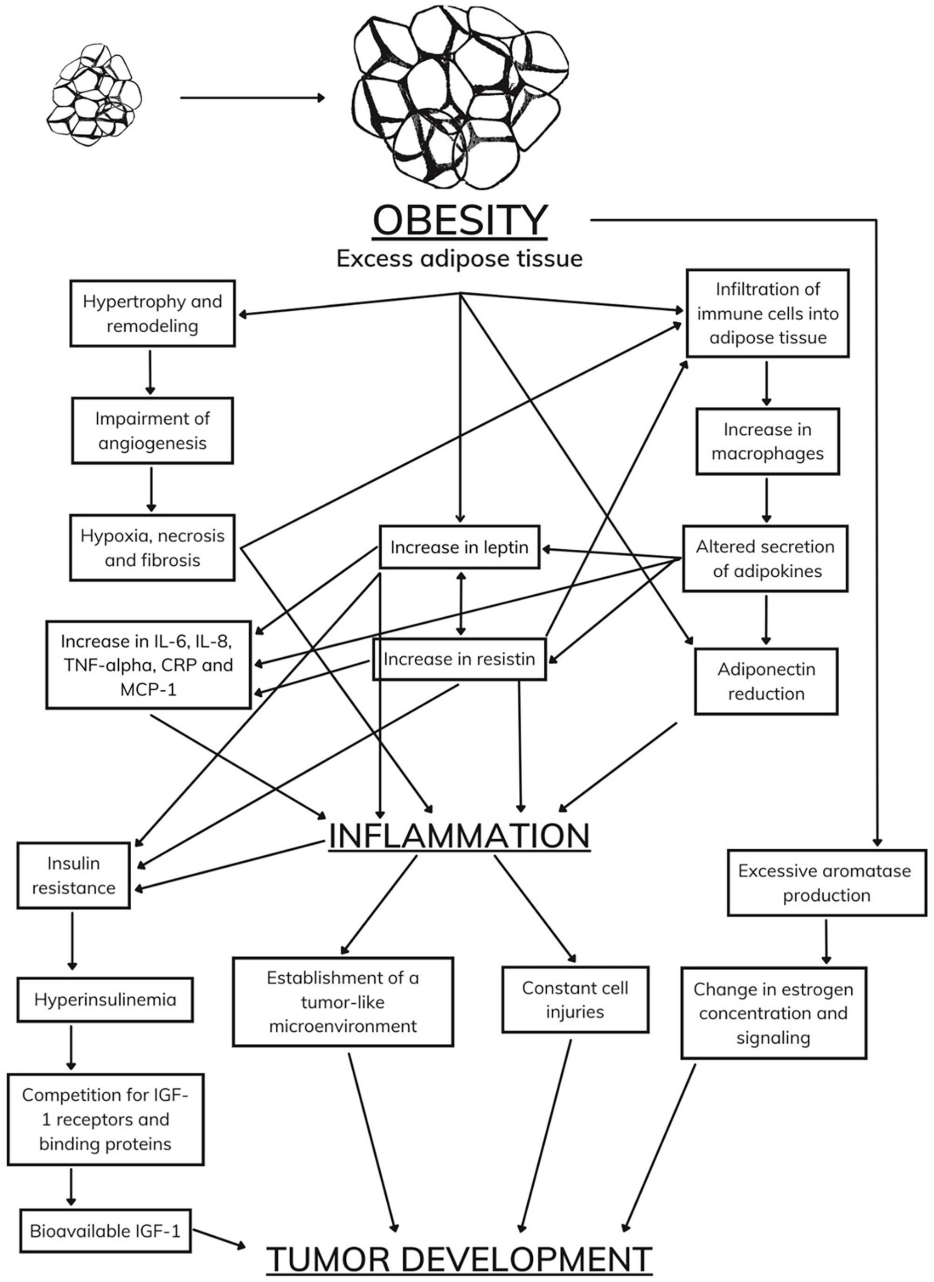
Body reaction to an increase in adipose tissue.

As in humans, some biomarkers have been shown to promote tumor development in animal models, such as an increase in the concentrations of IGF-1, leptin, and sex hormones and a decrease in the concentration of adiponectin (159). Despite scientific advances, there is still a considerable gap in the complex and comprehensive relationship between obesity and tumor development (Figure 2).

## Discussion

Although obesity is known to be linked to type 2 diabetes in humans, in dogs, diabetes is generally due to the lack of insulin-producing pancreatic β cells. This is similar to type 1 diabetes in humans, which is not caused by obesity. However, obesity decreases insulin sensitivity in dogs, and it is possible that it may also reduce the control of diabetes (18, 20, 60, 160). In addition, Thengchaisri et al. (161) evaluated 44 obese dogs and found that abdominal obesity was associated with heart disease. Overall, biological mechanisms linking obesity to cancer include insulin resistance and abnormalities of the IGF-1 system and signaling; sex hormone biosynthesis and pathway; subclinical chronic low-grade inflammation and oxidative stress; alterations in adipokine pathophysiology; ectopic fat deposition; microenvironment and cellular perturbations including vascular perturbations, epithelial-mesenchymal transition, endoplasmic reticulum stress, and migrating adipose progenitor cells; disruption of circadian rhythms and other factors with potential significance such as the altered intestinal microbiome; and mechanical impairment factors in obesity and cancer (150).

There are not many studies on the relationship between obesity and cancer in dogs. The first study to correlate obese with cancer development in dogs was conducted in 1989 (21). Glickman et al. investigated the relationship between chemical and insecticide exposure, obesity, and naturally occurring transitional cell carcinoma (TCC). Through interviews and questionnaires, they investigated the dogs' body composition 1 year before diagnosis and whether they were exposed to such factors. They observed that the risk of developing TCC of the bladder was greater in obese dogs regardless of exposure to topical insecticides (21). Two years later, Sonnenschein et al. (22) identified that, among neutered female dogs, obesity that developed in early life could increase the risk of developing mammary gland tumors. In the same way as the previous study, this was carried out using a questionnaire and also had the limitation of verifying body conditions retrospectively. Similarly, Weeth et al. (23) identified a possible association between obesity and the development of mast cell tumors.

In intact female dogs, the most common neoplasm is the mammary gland tumor, with an incidence of malignancy of approximately 50% of diagnosed tumors cases (162). Owing to the similarity between canine mammary gland tumors and breast cancer in women, female dogs serve as animal models for future investigations in human oncology (163). Many studies have been conducted on obese female dogs diagnosed with different types of mammary gland neoplasms. Currently, progress in the understanding of the pathophysiology of obesity and tumors, as well as the influence between them, has allowed studies to be carried out that seek to identify more specific changes. According to a study published by Queiroga et al. (164), prolactin and steroid hormones are related to the growth of canine breast cancer, having possible autocrine and paracrine roles in its maintenance. On the other hand, the loss or decrease of gonadal hormones as a result of gonadectomies can increase the incidence of obesity, endocrine disorders, and cancer, among other diseases in animals (165). In addition to the functions already described, peripheral adipose tissue is responsible for secreting aromatase, which converts androgen hormones into estrogens (152). Perez-Alenza et al. (162) verified that increased age, non-castration, ovariectomy after 2.5 years, and progestogen treatment could increase the risk of mammary neoplasia in female dogs. Obesity early in life and homemade food (with low chicken content, but rich in beef and pork) were also associated with an increased incidence of mammary tumors.

In a study carried out by Lim et al. (24), the objective was to determine the relationship between the expression of adipokines, such as aromatase, leptin, and IGF-1 receptors, in mammary gland carcinomas, based on the BCS of female dogs. A total of 56 tumors were compared for the expression of adipokines, age at tumor development, histological grade, lymphatic invasion, degree of tumor necrosis, and the presence of estrogen and progesterone receptors. A priori, overweight and obese female dogs presented at earlier ages of development of mammary gland carcinomas compared to female dogs with ideal weight. Mammary tissue from dogs with mammary gland tumors in dogs show much higher aromatase expression than healthy mammary tissues (25). In this study, 80% of tumors with aromatase expression were observed in obese or overweight dogs. In summary, it was not possible to correlate the BCS with the expression of tumor estrogen and progesterone receptors, but they were associated with the expression of leptin, aromatase, and IGF-1 receptors. As in humans, aromatase seems to play a fundamental role in the development of mammary gland carcinomas in obese and overweight female dogs.

In contrast, Shin et al. (166) found no correlation between aromatase concentration, being overweight, and obese in dogs. However, its expression was associated with tumor expression of prostaglandin 2 (PGE2) and leptin. PGE2, not yet described in the present review, has a carcinogenic function by influencing the tumor microenvironment through its interaction with abnormal receptors, thus modulating the tumor-associated immune response, suppressing apoptosis, stimulating angiogenesis, and promoting metastasis (167, 168). Similar to PGE2, estrogen and progesterone play a significant role in the growth and invasion of breast tumors in women (169). Shin et al. (166) identified an exacerbated tumor expression of PGE2 in obese and overweight female dogs, which indicates major importance in the tumorigenesis of mammary gland tumors in dogs, as well as in humans. Furthermore, although not confirmed, there is evidence that being overweight increases the secretion of aromatase, insulin, and IGF-1, stimulating carcinogenesis in mammary gland tumors in female dogs (170).

In a similar study, Lim et al. (171) identified that overweight or obese female dogs developed mammary gland carcinomas earlier than female dogs at their ideal weight. Furthermore, they identified that the incidence of metastasis through lymphatic channels was higher, and that they developed tumors at a more advanced stage when compared to tumors in female dogs with ideal body weight. The development of mammary gland tumors at earlier ages and in more advanced stages in obese or overweight female dogs is in agreement with a similar study by Lim et al. (24). In addition, obese or overweight female dogs had a greater number of tumors with the absence of adiponectin and a greater concentration of infiltrating macrophages. Therefore, they associated the presence of adiponectin and macrophage infiltration in tumor masses with tumor staging and lymphatic infiltration. The absence of adiponectin and the greater number of infiltrating macrophages were positively correlated with more advanced, grade III tumors with lymphatic invasion; among tumors with adiponectin expression, the percentage of grade III mammary gland carcinomas was lower (171). Thus, these results imply that adiponectin may have a protective function by slowing tumor development, as already observed in humans. Additionally, obesity-induced macrophage recruitment may stimulate the development, growth, and metastasis of mammary gland carcinomas in female dogs. Tesi et al. (172) confirmed the relationship between obesity and being overweight in female dogs with the development of more advanced mammary gland carcinomas. The primary objective was to relate adiponectin expression with parameters such as tumor size, histological grade, degree of lymphatic invasion, and body score index; however, no correlations were observed.

Elevated resistin concentrations have been associated with obesity and are excellent markers for the prognosis of breast cancer (13, 162, 173). A study carried out by Nicchio et al. (174) evaluated the serum concentration of resistin in female dogs with or without mixed mammary carcinoma and the relationship between adipokines and obesity, tumor, and patient survival. They were able to elucidate that a resistin concentrations three-fold higher resistin concentrations in obese females compared to ideal-weight females, which corroborates with findings of serum resistin in humans with obesity. Furthermore, obese and overweight female dogs were found to have higher levels of resistin and mixed mammary carcinomas with greater malignancy potential, as demonstrated by increased cell proliferation and shorter survival times. Leptin and resistin are thought to exhibit similar behaviors (175). Leptin is correlated with the modulating the action of steroid hormones (24) and with the stimulation of metastases in mammary gland tumors (175).

Despite recent discoveries, studies on this topic have faced many difficulties. In a study by Romano et al. (176), although the objective was to associate overweight or obese dogs with shorter survival times when diagnosed with lymphoma or osteosarcoma, this could not be confirmed. It has been hypothesized that this could either be due to a greater number of overweight dogs compared to obese dogs in the study, which may have been a limiting factor for matching obese dogs to dogs with ideal weight; because the harmful effects of obesity may differ between cancer types; or due to the difference in sampling used in comparison with studies performed in humans, which present considerably higher numbers of cases (177). Most studies have investigated the relationship between obesity and the development of mammary gland tumors in female dogs. Despite the many concrete correlations that indicate possible paths for the identification of biological markers, there is still a long way to go. In veterinary medicine, considering the extremely small number of published scientific articles on the subject portrayed in the present study, this path is even more arduous. However, if we already know that there is a link between obesity and cancer in dogs, cancer-preventive measures may include weight loss with dietary modifications to control chronic inflammation and other obesity effects (177).

## Perspectives

Since a relationship between obesity and cancer has been found in both humans and dogs, it is reasonable to propose that avoiding obesity or treating obese dogs with weight loss could reduce the risk of developing cancers. German et al. (3) indicated the urgent need to increase awareness of companion animal obesity to avoid its consequences. Linder et al. (178) collected data from three pet festivals in different locations across the US; visitors over 18 years of age with their dog present were invited to participate in a dog-and-owner study. The body mass index (BMI) was measured in both humans and dogs with BCS. Owner BMI was positively correlated with dog BCS, supporting a possible association between overweight status of dogs and their owners. There are several reasons for this, including behavioral causes (eating too much and lack of exercise), misinformation, the psychosocial impact of the human-animal bond, and environmental exposures. An increase in physical activity and dietary changes is one of the measures to reduce obesity. Weight loss programs for canines are of utmost importance as veterinary diets must be used. These are based on the establishment of a negative energy balance obtained using mathematical equations (179). Despite the caloric restriction, as stated by Olivindo et al. (180), diets formulated for the management of obesity should not cause nutritional deficiencies; hence, the importance of consultation with nutrition specialists.

To ensure an effective weight-loss program, it is important to evaluate each case individually, as the rate of weight loss can be influenced by many factors. Vendramini et al. (181) evaluated the entire weight loss process of 77 obese dogs of different ages, sizes, breeds, and fertility status. By estimating the average calorie restriction requirement, they were able to identify that spayed females and mixed-breed dogs needed fewer calories during a weight loss program, which reduced the weight loss rate of these animals. Another variable that may be related to the reduction in the efficiency of weight loss is the intestinal microbiome. Obesity is associated with alterations in the gut microbiome in humans, mice, and dogs, leading to gut dysbiosis (182–184). A study by Kieler et al. (185) indicated that the reduction of *Megamonas* and Ruminococcaceae populations in the gut microbiome of obese dogs resulted in a higher rate of weight loss. As these bacteria are producers of acetic and propionic acid, the hypothesis is that the organism in a negative energy balance adapts to use short-chain fatty acids for energy. Macedo et al. (186) evaluated the effects of weight loss on fecal microbiota in 10 obese female dogs and compared the results with those of lean dogs. The specific reduction of the *Megamonas* genus after weight loss was similar to that in a previous study and, in general, it was possible to confirm that the weight loss program can reverse changes in the microbiome of obese dogs.

Gut dysbiosis has been associated with the development of acute diarrhea and inflammatory bowel disease in dogs (187, 188). Furthermore, it appears to play an important role in the carcinogenesis of certain neoplasms. Gavazza et al. (189) measured the microbial populations in fecal samples from 12 dogs with multicentric lymphoma and compared them with those of 21 healthy dogs. In general, there was a difference in the dysbiosis index between the dogs with cancer. In a similar study, Herstad et al. (190) observed changes in the profile of fecal microbiota and intestinal mucosa of dogs with colorectal tumors and healthy dogs. Dysbiosis was not restricted to the tumor tissue because the adjacent non-tumor mucosa presented the same population characteristics as the tumor tissue. This indicates that the differences between tumor-bearing and healthy dogs are not only the result of local tumor disturbances. Despite the differences found in these studies, it was not possible to conclude whether changes in the microbiota were the cause or effect of cancer. The human microbiome's role in carcinogenesis is still a mystery as well. A recent consensus that brought together 18 experts in the field categorized this influence as unproven; however, most collaborators agreed with the hypothesis that the microbiome works together with environmental factors and genetic predispositions in the development of cancer (191). The involvement of obesity as an environmental factor associated with the microbiome is an interesting frontier of research and may elucidate several mechanisms that remain undiscovered.

The omics sciences can contribute to new discoveries, especially metabolomics, whose objective is to measure all or a large part of an organism's metabolites (192). In this way, it can elucidate the differences in the metabolic profile and possible metabolic adaptations of an organism with a certain disease. The information obtained allows for a better understanding of the stage of the disease and the oxidative stress caused by it; consequently, the data obtained usually provide better subsidies for a more effective therapeutic approach (193–195). Kawabe et al. (195) sought to determine the plasma metabolic profile of dogs diagnosed with melanoma to identify possible biomarkers for the diagnosis and prognosis of the disease. Samples from nine healthy dogs were compared with those from 32 dogs diagnosed with melanoma. Significant differences in metabolites were found in dogs with melanoma, such as three amino acids (threonine, proline, and serine), citric acid, fatty acids, and glycerol. These results suggest that metabolism is increased in melanoma, as has been suggested for other neoplasms (196, 197). A recent study performed by Vendramini et al. (198) evaluated the blood serum metabolic profile of obese dogs, control dogs, and dogs undergoing a weight loss program, and approximately 20 metabolites were identified by nuclear magnetic resonance of the blood serum of the animals. The metabolomic evaluation differed between obese dogs and animals with ideal BCS. Furthermore, weight loss results in metabolomic profiles that are similar to those observed in lean animals. The metabolites that were different between the groups were glucose, lactate, glutamine, acetone, arginine, alanine, and citrate for the grouping of healthy animals (control and after weight loss) and lipids, cholesterol, and branched-chain amino acids for obese animals. Another recent article was carried out by Carlos et al. (199), which consisted of a review of the current applications of metabolomics in domestic dogs. This review included 38 articles using canine metabolomics as a tool and demonstrated that several diseases can be identified with the determination of biomarkers and/or the understanding of the mechanisms of action in the metabolism of dogs.

## Conclusion

Currently, the available literature supports white adipose tissue as an endocrine organ. Inflammatory, endocrine, and metabolic disorders triggered by obesity lead to alterations in the endocrine function of adipose tissue which are synergistic with mechanisms that promote carcinogenesis. Although still poorly understood, adipokines clarify the effects of obesity in dogs and elucidate their relationship with carcinogenesis. In this way, it is possible to highlight the mechanisms of action of leptin, resistin, adiponectin, aromatase, IGF-1, and pro-inflammatory cytokines in cancers to characterize new associations between obesity and other neoplasias.

## Author contributions

Writing—original draft: PM, TV, MP, RZ, AA, VO, and MB. Writing—review and editing: PM, TV, JD, MD, and MB. All authors have read and agreed to the published version of the manuscript.

## Conflict of interest

The authors declare that the research was conducted in the absence of any commercial or financial relationships that could be construed as a potential conflict of interest.

## Publisher's note

All claims expressed in this article are solely those of the authors and do not necessarily represent those of their affiliated organizations, or those of the publisher, the editors and the reviewers. Any product that may be evaluated in this article, or claim that may be made by its manufacturer, is not guaranteed or endorsed by the publisher.
